# The effect of health literacy intervention on adherence to medication of uncontrolled hypertensive patients using the M-health

**DOI:** 10.1186/s12911-023-02393-z

**Published:** 2023-12-15

**Authors:** Maryam Karami, Hossein Ashtarian, Mojgan Rajati, Behrooz Hamzeh, Fatemeh Rajati

**Affiliations:** 1 Student Research Committee, Kermanshah University of Medical Sciences, Kermanshah, Iran; 2 Department of Health Education and Health Promotion, School of Health, Kermanshah University of Medical Sciences, Kermanshah, Iran; 3 Department of Obstetrics and Gynecology, School of Medicine, Kermanshah University of Medical Sciences, Kermanshah, Iran; 4 Research Center for Environmental Determinants of Health, Health institute, Department of Health Education and Health Promotion, School of Health, Kermanshah University of Medical Sciences, Kermanshah, Iran

**Keywords:** Hypertension, Health literacy, Mobile Applications

## Abstract

**Background:**

Given that patients’ medication adherence is regarded as the major part of disease control and improving health literacy can be effective in promoting adherence to healthy behaviors, the present study aimed to investigate the effect of health literacy intervention based on the medication adherence among uncontrolled hypertensive patients using mobile health (M-health).

**Methods:**

An interventional study with a quasi-experimental design, was conducted on 118 uncontrolled hypertensive patients. Participants were randomly divided into the intervention (n = 59) and control (n = 59) groups using blocked randomization. In the intervention group, a mobile health (M-health) program was designed using programmed instruction to improve patients’ health literacy over a period of 3 months. Data was collected by administering health literacy and medication adherence questionnaires to participants before and after the intervention. The analysis involved using the independent sample t-test to compare the variables before and after the study.

**Results:**

Before the intervention, the total score of health literacy was 33.34 and 33.14 in the intervention and control groups, respectively. After the intervention, it increased to 40.36 and 34.20 in the intervention and control groups, respectively, which was statistically significant in the intervention group (p = 0.01). Moreover, the medication adherence score of the intervention group significantly increased after the intervention. Both systolic and diastolic blood pressure decreased in the intervention group. However, it should be noted that the decrease in systolic blood pressure by 148.98 was statistically significant, while the decrease observed in diastolic blood pressure in the intervention group was not statistically significant (p = 0.08).

**Conclusion:**

The application of programmed instruction through M-Health has shown a positive effect on the health literacy of uncontrolled hypertensive patients. In addition to detecting and treating patients, it is important to prioritize the improvement of health literacy in terms of medication adherence and the adoption of healthy behaviors.

## Background

Hypertension is considered as a growing health problem and the third cause of death in the world, as about 31% of adults over 18 years old in America suffer from hypertension [1]. Hypertension is defined as systolic blood pressure ≥ 140 mmHg or greater and diastolic blood pressure ≥ 90 mmHg or greater [2]. This disease is recognized as one of the most common risk factors for cardiovascular diseases, leading to myocardial infarction, brain accidents, heart failure, vision disorders, kidney failure, and premature death [3]. According to a recent systematic study conducted in Iran, the prevalence of hypertension is variable in Iran and its overall prevalence is estimated to be 22% [4].

One of the main determinants of blood pressure control is self-care behavior, including regular control of blood pressure, reducing salt intake, not using tobacco, physical activity, avoiding stress, healthy nutrition, losing weight, and taking medicines prescribed by the physician timely [5]. In the same vein, non-adherence to medication is one of the problems of hypertensive patients. Medication adherence in hypertensive patients includes adherence to medicinal and non-medicinal methods that the patient should implement [6].

Ravansar cohort study in Iran demonstrated that 15.7% of 10,040 people aged 35 to 65 have hypertension and only 53% of them are successful in controlling their disease and 73% of them receive antihypertensive drugs [7]. A meta-analysis study in Iran revealed that blood pressure is not controlled in more than 63% of hypertensive patients [4, 8–10].

Inadequate adherence to medication leads to increased morbidity, mortality, and health care costs, as well as frustration among patients and health care providers [11, 12]. Non-adherence to medication limits the effectiveness of preventive strategies and leads to a significant increase in cardiovascular complications [13].

Health literacy leads to more awareness of health issues and consequently, better self-care [14, 15]. The World Health Organization (WHO) introduced health literacy as one of the most important determinants of health [16]. The systematic review studies illustrated that the criteria of defining literacy vary in different studies. Health literacy is the capacity to obtain, process, and understand basic information and services needed for making appropriate decisions in the field of health [17, 18].

The American Medical Association (AMA) divided the field of health literacy into the cultural and conceptual knowledge, print health literacy (writing and reading skills), oral health literacy (listening and speaking), and numeracy.

Low health literacy makes people unable to find and use the health information and cares they need [19]. Further, about 40–80% of provided medical information given by medical staff is reported to be immediately forgotten [20]. Studies show adequate health literacy is a determinant factor to control hypertension among hypertensive patients [21, 22]. Further, health literacy promotion interventions influence patients’ medication adherence [23]. There is a wide range of successful applications of new technologies, especially targeted interventions of mobile health (M-health) application in controlling and lowering blood pressure and increasing the health knowledge level and medication adherence [24].

Based on the results of the studies, the use of m-health application among patients with heart failure, hypertension, and diabetes improves the efficiency of the health care system, quality of life, and medication adherence and reduces costs [25]. However, currently these technologies are applied to change the behavior of Iranian patients in a limited way.

Programmed instruction was introduced by Skinner in America (1954), professor of psychology at Harvard University. Programmed instruction is an individual instruction method, dividing the content into small section and presenting them gradually and in regulated steps. Individuals study these materials step by step and after completing each step and before moving to the next step, they are tested to evaluate their understanding [26]. Since the programmed instruction provides reinforcement when the learner illustrates the desired behavior, this method can be an appropriate way to provide feedback to people and help interactive learning.

Considering that hypertension is usually associated with asymptomatic symptoms, the person may forget to take their medicine regularly and perform healthy behaviors. Therefore, the programmed instruction is considered as an appropriate method for these patients, since this issue is partially due to the low health literacy of the patients. In this method, the educational material is taught to the person step by step, and after learning the content of each step, the person moves to the next step. Given that this method may be associated with trial and error, it stabilizes learning in the patient. However, interventions to improve health literacy in different domains, including print health literacy and functional literacy, are challenging [27]. Therefore, it seems that the nature of programmed instruction using M-health technology is effective in improving people’s health literacy, adherence to medication and healthy behaviors, and blood pressure control. The present study aimed to investigate the effect of health literacy intervention based on M-health on the medication adherence behavior of hypertensive patients using programmed instruction.

## Methods

### Study design and procedure

This study was a quasi-experimental design. It was conducted on 118 hypertensive patients referred to a health center in Kermanshah, Iran from October to February 2021. Patients were selected using convenient sampling method based on the inclusion and exclusion criteria and randomly divided into the intervention (n = 59) and control (n = 59) groups. For this study, 118 individuals were allocated into two groups using block randomization with the assistance of random allocation software (https://random-allocation-software.software.informer.com/download/?caa49a). The allocation was done in 15 blocks of 8, with 4 individuals assigned to the intervention group and 4 individuals assigned to the control group in each block. All patients completed the informed consent form. Based on the study results of Hacihasanoglu’s et al. (2011), education increases the patient’s medication adherence by 10–32% [27]. Therefore, the sample size was estimated using the following formula to be at least 59 patients, considering anapproximately 95% confidence interval and a power of 90%, along with the assumption of 10% attrition in each group:

$${n_1} = {n_2} = \frac{{({p_1}(1 - {p_1}) + {p_2}(1 - {p_2})){{({Z_{1 - \frac{\alpha }{2}}} + {Z_{1 - \beta }})}^2}}}{{{{({p_1} - {p_2})}^2}}}$$

When n_1_ and n_2_ represent the sample size in each group, ρ denotes the rate of medication adherence, α was considered as 0.05, and β is set at 90%.

The inclusion criteria were patients aged over 30 years with reading and writing literacy, who have been diagnosed as hypertensive patients in the health center but failed to control their hypertension (systolic blood pressure ≥ 140 mmHg and diastolic blood pressure ≥ 90 mmHg). All patients took medication for their hypertension. The exclusion criteria included people with underlying chronic diseases, including cardiovascular disease, diabetes, and cancer or pregnant women.

### Measurement

The data were collected using demographic characteristic questionnaire, hypertension-health literacy scale (HBP-HLS), and hypertensive patients’ adherence to medication, made by the researcher.

#### Demographic characteristic questionnaire

This questionnaire included the variables of age, gender, height, weight, body mass index, educational level, marital status, employment status, duration of hypertension disease, and regular use of medication. Further, systolic and diastolic blood pressure were measured twice based on JNC-8 and recorded [10].

#### Hypertension-health literacy scale (HBP-HLS)

The scale was developed by Kim et al. (2012) [28], which was translated and re-translated by the researcher according to the Beaton guideline [29]. The questionnaire composed of the oral literacy (speaking and understanding speech), print literacy (reading, writing, and understanding written language), and functional literacy (performing a specific task) subscales. This questionnaire focuses on measuring print literacy and functional literacy of patients.

In the first part of the questionnaire to measure print literacy, the patient pronounces 30 words in three levels and the difficulty of the words is ascending in each level. Each word was scored as correct (1) and incorrect (0).

In the second part of the questionnaire, The test of functional health literacy in adults (TOFHLA) [30] and Newest Vital Sign (NVS) [31] tools with 13-itme were used to measure functional literacy of hypertensive patients. TOFHLA measures numeracy and reading comprehension skills. The calculation subscale measures a person’s ability to understand and act based on the recommendations given by doctors and health educators. This subscale includes three health explanations or orders, which are provided to the subjects in the form of cards. Further, the NVS includes a ramen (popular noodle among Koreans) nutrition label with six items, assessing the ability to interpret nutritional information and reading, comprehension, and calculation skills that are necessary to understand health information well. In this questionnaire, 7 items from TOFHLA and 6 items from NVS were given. The items were scored as correct (1) and incorrect (0), and their total score ranges from 0 to 13. A higher score indicates higher health literacy. The content validity index (CVI) and content validity ratio (CVR) values of the questionnaire were obtained as 0.87 and 0.60, respectively. The reliability of the questionnaire was 0.87 using Kuder Richardson Formula. TOFHELA was used and translated to Persian by Javadzadeh et al. [32].

#### Questionnaire of adherence to medication of hypertensive patients

A questionnaire was developed in a study to measure the medication adherence of hypertensive patients. For this purpose, a mixed method study was designed in two phases, including the development of the questionnaire and the evaluation of psychometrics. In the first phase, a qualitative study was designed with the steps recommended by Waltz et al. [33] using the conventional content analysis method to explore the concepts of medication adherence among hypertensive patients. The data were collected through semi-structured interviews until the data saturation stage and then, analyzed. The questionnaire items were designed on a 5-point Likert scale (never, rarely, sometimes, often, and always) based on the results obtained from qualitative interviews using available literature and revised by an expert panel. In the second phase, after evaluating the content validity, the expert panel was asked about difficulty, wording, grammar, and comprehensibility to modify the questionnaire based on their opinions. Then, the essentiality and the relevance of each item were asked from the expert panel. The content validity ratio (CVR) was considered as 0.42 based on the Lawche Table [34].

Item’s content validity index (I-CVI) by dividing the number of experts (18 individuals) who had given that item the score of 3 or 4 by the total number of experts. The items with CVI > 0.79 were considered appropriate, those with CVI = 0.7–0.79 were revised, and the items with CVI < 0.7 were excluded [35].

Twenty hypertensive patients evaluated the face validity of the questionnaire qualitatively and quantitatively. Difficulty and relevancy of each item was assessed in the qualitative face validity stage. The items’ impact score was calculated in quantitative face validity stage. The same hypertensive patients rated the importance of each item, scored between 1(unimportant) to 5 (very important). Impact score for each item was calculated using the following formula:

“Impact score = Frequency (%) × Importance”,

where “frequency” represents the number of patients rated the item 4 or 5, and “Importance” displays the average of the item on the 1–5 rating scale. The items with impact scores equal or greater than 1.5 were kept. Reliability analysis was employed using test-retest intra-class correlation index (ICC) by 30 eligible patients who asked to complete the questionnaire twice with two interval weeks. The ICC of the questionnaire was 0.83 (95% CI: 0.76–0.90, *P* < 0.001). The Cronbach’s alpha was calculated as 0.71, which was greater than the cut-off value of 0.7.

#### Preparing M-health application based on programmed instruction

In this research, an educational M-health application was prepared based on the protocol proposed by Sadegh et al. [36] to increase the health literacy of hypertensive patients. The educational material of hypertension was prepared based on the guidelines of the hypertension management in the American College of Cardiology/American Heart Association [37]. The educational material of M-health included the definition, classification, complications, and medications of hypertension, hypertension measurement, and diet, which were individually provided for people in separate sections using the programmed instruction. The educational material was divided into small sections and presented to the patients gradually and in regular sequence. Patients studied the materials step by step and after completing each step successfully and before entering the next step, they answered the test questions of each section to assess their understanding.

If the patient chooses the wrong answer, the application returns him to the previous stage to receive the education and study again to select another answer, as the person is guided to the correct answer and consequently, learning takes place. Accordingly, as this learning process progressed, it helped improve the health literacy of patients. Therefore, the educational content and M-health implementation method were designed based on the programmed instruction method and concepts about the printed and functional health literacy. The educational material and application program were designed and implemented by five specialists, including three health education specialists, one doctor, one nurse, and one IT in Health specialist during several sessions. The pilot evaluation of the application was done among ten patients before finalization, and the patients were asked to assess and give feedback on any ambiguity, difficulty, and incomprehensibility of the content or the performance of application. After brief changes, the initial version of the application was finalized. Then, the usability of the application was examined using the opinions of the health care system managers, and the final modifications were made based on the national guidelines for controlling and managing blood pressure, which is supervised by the health deputy in the universities of medical sciences.

### Interventions to improve health literacy

After preparing the application, the educational intervention was conducted in areas of health literacy, spanning across four sessions. Patients were also instructed to utilize the application at home. One effective approach to enhance health literacy in the domain of reading is to utilize plain language and avoid technical jargon when communicating health information to patients. In this regard, we employed physiological and anatomical terminologies, along with synonyms, definitions, and related pictures where applicable. Examples of such words include cardiovascular disease, hypertension, uncontrolled hypertension, systolic blood pressure, diastolic blood pressure, etc. We used these terminologies in certain scenarios, emergencies, and medical situations and asked patients to discuss and converse about them.

As visual representations can help clarify information and make it more accessible, we also provided visual materials such as diagrams, illustrations, and charts to enhance understanding. We also made efforts to engage patients in interactive activities, discussions, and role-plays to actively involve them in the learning process. This hands-on approach can enhance comprehension and retention of health information.

To improve patients’ understanding and consequently decision-making, we provided clear and accessible health information about hypertension and its management. We invited their families to create a supportive and non-judgmental environment where individuals feel comfortable asking questions and seeking additional information, which can promote active involvement in decision-making. Additionally, we utilized M-health as a shared decision-making tool by checking patients’ assignments completed in M-health during each session. If any issues were identified, we provided feedback and resolved them to improve patients’ adherence to medication.

### Statistical analysis

In this study, the statistical analysis involved the use of an independent t-test to assess the differences in quantitative variables between the intervention and control groups. The homogeneity of qualitative variables was evaluated using the Chi-square and Fisher’s exact test. Mean and standard deviation were used to report and describe the quantitative variables, while frequency and percentage were employed for qualitative variables. Levene’s test was conducted to check the equality of variances for the investigated variables, including health literacy score, medication adherence, and systolic blood pressure score, between the intervention and control groups. The null hypothesis assumed equal variances between the two groups. Subsequently, the independent *t*-test was used to determine the significance of the mean scores of the investigated variables between the control and intervention groups. Paired t test was also used to access the change within groups of intervention and control. The significance level was set at 0.05, and data analysis was performed using SPSS version 22 software.

## Results

The mean age of patients was 52.20 ± 15.55 years. The homogeneity of variables such as sex, education status, job, and marital status was assessed using the Chi-square test, which involved dividing the different levels of variables into two intervention and control groups. We used the Fisher’s exact test when more than 20% of the cells in the cross-tabulation had expected counts less than 5. The mean ± SD systolic and diastolic blood pressure were 149.13 ± 11.3 and 94.8 ± 12.4 mm Hg in the intervention group, respectively and 148.9 ± 16.3 and 94.8 ± 12.4 and 95.6 ± 7.4 mm Hg in the control group. The mean duration of hypertension was 6.63 ± 4.09 years, and the mean of BMI of the patients was 26.54 ± 3.84 kg/m^2^ in all participants (Table 1).

**Table 1:** The characteristic of patient’s demographic and clinical variables of 118 patients

Variables		Intervention (n = 59)	Control (n = 59)	*p-*value
**Sex** n (%)	Man	31 (26.3)	28 (23.7)	0.09*
	Female	22 (18.6)	37(31.4)	
**Education** n (%)	Primary level	36 (30.5)	16 (13.6)	0.46**
	Middle School	13(11.0)	4(3.4)	
	Diploma	3(2.5)	4(3.4)	
	Associate Degree	2(1.7)	0(0.0)	
	Bachelor’s degree	3(2.5)	2(1.7)	
	Master’s degree	2(1.7)	0(0.0)	
**Job** n (%)	housewife	26 (22.0)	35	0.05**
	Employee	3 (2.5)	1 (0.8)	
	Part time job	24 (20.3)	23 (19.5)	
	Other	3 (2.5)	3 (2.5)	
**Marital status** n (%)	Single	4(3.4)	5(4.2)	0.77*
	Married	47(39.8)	47(39.8)	
	Divorced	1(0.8)	0(0.0)	
	Deceased wife	7(5.9)	7(5.9)	
**Systolic blood pressure**^**a**^ (Mean ± SD)		149.13 ± 11.3	146.61 ± 9.5	0.69***
**Diastolic blood pressure**^**a**^ (Mean ± SD)		94.83 ± 12.4	95.59 ± 7.5	0.77***
**BMI (**Mean ± SD)		26.5 ± 3.8	25.8 ± 4.2	0.37***
**Duration of the morbidity**^**b**^**(**Mean ± SD**)**		6.63 ± 4.1	6.95 ± 4.6	0.16***

a:mmHg, b:year

*Chi-square test

** Fisher’s exact test

***independent sample *t*-test

The mean score of health literacy in the domain of reading was 25.17 ± 3.30 in the intervention group and 24.40 ± 6.60 in the control group before the intervention and changed to 26.89 ± 1.68 in the intervention group and 24.45 ± 6.57 in the control group after the intervention. Based on the results of the independent *t*-test, the mean score of health literacy in the reading level had a statistically significant change in the intervention group. However, the mean score did not change statistically in the control group (*p*-value = 0.80) (Table 2).

Before the intervention, the mean score of health literacy in the field of comprehension was 3.32 ± 1.99 in the intervention group and 3.25 ± 2.02in the control group, which reached to 5.1 ± 0.99 in the intervention group and 3.28 ± 2.03 in the control group after the intervention. The results of the independent *t*-test demonstrated that the mean score of health literacy regarding comprehension and understanding had a statistically significant change in the intervention group(*p*-value < 0.0001), and did not statistically change in the control group (*p*-value = 0.78) (Table 2).

The mean scores of health literacy in the domain of decision-making and use of health information was 4.48 ± 2.0 in the intervention group and 5.49 ± 1.63 in the control group before the intervention and after the intervention, increased to 6.56 ± 0.79in the intervention group and 5.52 ± 1.65 in the control group. The results of the independent sample *t*-test indicated that the mean scores of health literacy in the domain of decision-making and use of information had a statistically significant change in the intervention group and the mean scores did not change statistically in the control group (*p*-value = 0.001) (Table 2).

Before the intervention, the mean score of medication adherence was 7.90 ± 1.82 and 8.19 ± 1.93 in the intervention and control groups (*P* = 0.41), respectively and after the intervention, reached to 10.31 ± 0.81 and 7.95 ± 1.88 in the intervention and control groups. The results of the independent *t*-test revealed a statistically significant change in the mean score of medication adherence in the intervention group (< 0.0001), while we observed a statistically significant decrease in adherence of medication in the control group (*p*-value = 0.01) (Table 2).

The mean score of systolic blood pressure was 149.83 ± 17.46 in the intervention group and 146.31 ± 9.47 in the control group before the intervention and after the intervention, this rate changed to 148.98 ± 16.47 and 146.31 ± 9.47 in the intervention and control groups, respectively. The results of the independent sample *t*-test showed a statistically significant change in the mean scores of health literacy of systolic blood pressure in the intervention group (*p*-value = 0.024) and no statistically significant variation in the control group (*p*-value = 0.70) (Table 2).

Furthermore, the mean score of diastolic blood pressure was 107.80 ± 99.11 in the intervention group and 95.57 ± 7.48 in the control group before the intervention. After the intervention, this value changed to 107.80 ± 99.06 in the intervention group and 95.57 ± 7.48 in the intervention group. The results of the independent paired-test illustrated no statistically significantly difference in mean score of diastolic blood pressure within either intervention (*p*-value = 0.083) or also within control groups (*p*-value = 0.86) (Table 2).

**Table 2:** The comparison of the mean and standard deviation of the investigated variables before and after the intervention

Outcome variables/Groups	Health literacy(reading) (mean ± SD)		*P*-value**	Health literacy (comprehension and understanding) (mean ± SD)		*P*-value**	Health literacy (decision-making) (mean ± SD)		*P-*value**	Adherence to medication (mean ± SD)		*P-*value**	Systolic BP (mean ± SD)		*P-*value**	Diastolic BP (mean ± SD)		*P*-value**
	Before	After		Before	After		Before	After		Before	After		Before	After		Before	After	
**Intervention** **(n = 59)**	25.17 ± 3.30	26.89 ± 1.68	< 0.0001	3.32 ± 1.99	5.1± 0.99	< 0.0001	4.48 ± 2.0	6.56 ± 0.79	< 0.0001	7.90 ± 1.82	10.3 ± 0.81	< 0.0001	149.83 ± 17.46	148.98 ± 16.47	0.024	107.29 ± 99.11	107.80 ± 99.06	0.083
**Control** **(n = 59)**	24.40 ± 6.60	24.45 ± 6.57	0.8	3.25 ± 2.02	3.28 ± 2.03	0.78	5.49 ± 1.63	5.52 ± 1.65	0.68	8.19 ± 1.93	7.95 ± 1.88	0.01	146.31 ± 9.47	146.61 ± 9.57	0.70	95.57 ± 7.48	95.59 ± 7.49	0.86
***P*** -value*	0.42	< 0.0001		0.85	< 0.0001		0.058	< 0.0001		0.41	< 0.0001		0.21	0.31		< 0.0001	< 0.0001	

* Independent sample *t*-test (between group analysis)

** Paired *t* -test (within group analysis)

## Discussion

According to the findings of the present study, the intervention based on programmed instruction led to an improvement in the health literacy of patients in the areas of reading, understanding and comprehension, and decision-making. It also resulted in an increase in the average systolic blood pressure and medication adherence in the intervention group than control group.

This study employed plain language, physiological and anatomical terminologies, visual aids, and interactive activities to engage patients in the learning process. The study found that the intervention improved patients’ understanding of hypertension and its management, and promoted active involvement in decision-making. Additionally, the use of M-health as a shared decision-making tool improved patients’ adherence to medication.

Having reading skill in the health literacy contributes patients to read and understand the concepts and instructions written on medicine bottles, appointment slips, and other essential health related materials. Some studies defined health literacy with great focus on the domain of reading.

Therefore, print literacy included reading and writing (the ability to decode letters and sound out words) has dominated the concept in health literacy so far. The focus on print literacy has yielded profound insights into difficulties and barriers linking literacy skills to health outcomes [38].

The results of the present study are consistent with the findings of previous studies. Peyman et al. (2016) reported a significant increase in the mean score of health literacy among patients after an educational intervention [39]. Similarly, Zhuang et al. (2016) demonstrated the significant role of educational programs in improving health literacy in China [40]. Therefore, new educational methods can be effective in improving low health literacy. Individuals with low health literacy are less likely to understand written and spoken information provided by health professionals and may struggle to follow instructions, leading to higher rates of hospitalization and referral to physicians [41].

In the present study, the mean score of the comprehension of the patients improved after the intervention, representing the effect of the intervention on the health literacy of the patients. Indeed, it is expected that hypertensive patients who have a better understanding of the etiology, mechanism, and complications of the disease will be more likely to adhere to their medication and experience fewer complications. Improved health literacy can empower patients to make informed decisions about their treatment, engage in self-management strategies, and effectively communicate with healthcare providers. This can ultimately lead to better medication adherence and improved health outcomes in hypertensive patients.

Adherence to healthy behaviors depends on patients’ understanding of their condition, treatment, and the benefits of lifestyle modification. The self-regulation model of illness perception suggests that the cognitive responses patients develop about their illness can influence their choice and appraisal of coping strategies [42]. Kalantari et al. (2012) demonstrated that the belief in the disease severity, disease outcomes, sever symptoms, poorer self-care and worry about the disease are associated with the lower quality of life [43]. In another study, Rajpura et al. (2014) reported a positive relationship between understanding of the disease and positive beliefs about treatment among the elderly with hypertension with high medication adherence [44]. In the study of Chen et al. (2011), the perception of the disease was associated with more medication adherence among patients [45]. Further, Ross et al. (2004) illustrated that disease perception and beliefs about hypertension are predictors of patients’ medication adherence [46]. Based on the study results of Kang et al. (2015), patients with better perceived health status are more likely to adhere to the treatment regimen [47]. In our study, improving health literacy probably helped patients to obtain, process, and understand health information more easily and make informed decisions, and consequently, increases their medication adherence.

In line with the results of the present study, Inglis et al. (2016) introduced M-health as a tool for the education of heart failure patients and reported that the use of M-health to manage heart failure detects and reduces symptoms, prevents hospitalization, improves quality of life, increases patient knowledge and self-care behaviors, preventing disease exacerbation, and helps to adhere to medication [25]. In the present study, the mean score of medication adherence of the patients increased in the intervention group compared to the control group, which was statistically significant and indicates the effect of the educational intervention of health literacy using mobile technology on the medication adherence of hypertensive patients. The study of Kilic et al. (2020) demonstrated a significant and positive relationship between the health literacy level and medication adherence among hypertensive patients, which is consistent with the results of the present study [49]. Wannasirikul et al. (2016) reported that health literacy has no direct effect on the medication adherence of hypertensive patients [50]. Since, in managing patients with uncontrolled hypertension, practitioners may only suspect that the drugs prescribed are not in accordance with the most up-to-date guidelines, an important issue such as health literacy as a factor influencing medication adherence should not be neglected [51]. However, to ensure their accuracy and effectiveness, it is important to validate these mobile applications by comparing them to other validated m-health applications, just as we do with health-related questionnaires.[52].

Programmed instruction is an individual educational method, dividing subject materials into small units and presenting them gradually in a sequential order. People study these materials step by step, and after completing each step and before entering the next step, they answer the test questions to assess their understanding and the health literacy of the person is improved in this way. Since hypertension with hidden nature is considered as a risk factor for cardiovascular disease and a predictor of cardiovascular disease mortality, the nature of hidden symptoms of hypertension can reduce the patients’ medication adherence, as hypertensive patients may experience few physical symptoms, leading to non-medication adherence. Mobile phone technology (M-health) can play a key role in controlling hypertensive patients’ health. Therefore, it is possible to reduce costs and the number of hospitalizations, and ultimately, empower patient without direct supervision of healthcare personnel using M-health. Programmed instruction in the electronic platform allows the patient to read the educational materials many times and benefit from the categories of reading, practice, and repetition. These findings suggest that utilizing an application and a hands-on approach can enhance health literacy and improve patient outcomes.

### Limitations

This study was some limitations. First, we recruited patients from health centers and no detailed information was available regarding the compatibility of their drugs with the updated guidelines. Second, this study failed to control diastolic blood pressure. It seems antihypertensive therapy fail to reduce diastolic blood pressure under 100 mmHg. Although there was a statistically significant difference in mean score of the systolic blood pressure after intervention within the intervention group compared to control group, this was not clinically important. It is important to note that our study comprised of hypertensive patients who were also overweight. The intervention primarily aimed to improve health literacy, but did not encompass all aspects of a healthy lifestyle for overweight patients. Therefore, incorporating lifestyle modifications in addition to the intervention may yield better results. Therefore, interventions based on improving health literacy should be considered as an important tool alongside lifestyle modification programs for hypertensive patients. Third, patients with unsuccessful hypertension control visited physician likely more frequently than patients with controlled hypertension as they are more relaxed and did not seek medical visits. So, the result of this study may not be generalizable to other patients. Further studies can expand upon on current work to more clarify the relationship between health literacy and other health outcomes.

## Conclusion

The results of the present study indicated that applying programmed instruction using the M-health has a positive effect on the health literacy of uncontrolled hypertensive patients and improves their medication adherence. Therefore, the health education programs with an emphasis on the use of programmed instruction and M-health methods are needed in promoting health literacy. In order to increase individuals’ health literacy level, health system employees should be trained on using programmed instruction and the key role of mobile technology in controlling patients’ health. Further, health policy makers should consider health literacy as one of the most important tools to improve self-care and formulate the applicable and relevant programs and patterns at the community.

## Data Availability

The data that support the findings of this study will be available by corresponding author upon request.

## Abbreviations

M-health: Mobile health
SBP: Systolic blood pressure
DBP: Diastolic blood pressure
CVI: content validity index
CVR: Content validity ratio
JNC 8: The eighth joint national committee
HBP-HLS: Hypertension-health literacy scale
TOFHLA: Test of functional health literacy in adults
NVS: Newest vital sign

## References

[1] Rana J, Oldroyd J, Islam MM, Tarazona-Meza CE, Islam RM (2020). Prevalence of Hypertension and controlled Hypertension among United States adults: evidence from NHANES 2017-18 survey. Int J Cardiol Hypertens.

[2] Williams B, Mancia G, Spiering W, Agabiti Rosei E, Azizi M, Burnier M (2018). 2018 ESC/ESH guidelines for the management of arterial Hypertension. Eur Heart J.

[3] Visseren FLJ, Mach F, Smulders YM, Carballo D, Koskinas KC, Bäck M (2021). 2021 ESC guidelines on Cardiovascular Disease prevention in clinical practice. Eur Heart J.

[4] Afsargharehbagh R, Rezaie-Keikhaie K, Rafiemanesh H, Balouchi A, Bouya S, Dehghan B (2019). Hypertension and Pre-hypertension among Iranian adults Population: a Meta-analysis of prevalence, awareness, treatment, and control. Curr Hypertens Rep.

[5] Gangi Susan, Peyman Nooshin, Meysami Bonab Soheyla,Esmaily Habibollah GS. Effect of Self-Care Training Program on Quality of Life and Health Literacy in the Patients with Essential Hypertension. Med J Mashhad Univ Med Sci 60:792–803.

[6] Bernal DDL, Stafford L, Bereznicki LRE, Castelino RL, Davidson PM, Peterson GM (2012). Home medicines reviews following acute coronary syndrome: study protocol for a randomized controlled trial. Trials.

[7] Rajati F, Hamzeh B, Pasdar Y, Safari R, Moradinazar M, Shakiba E (2019). Prevalence, awareness, treatment, and control of Hypertension and their determinants: results from the first cohort of non-communicable Diseases in a kurdish settlement. Sci Rep.

[8] Arabshahi A, Qom University of Medical Sciences, Gharlipour Z, Qom University of Medical Sciences, Hosseinalipour SA et al. Qom University of Medical Sciences,. Assessment of adherence to self-care behaviors in hypertensive patients in Qom. Qom Univ Med Sci J. 2020;14:55–66.

[9] Pawloski PA, Asche SE, Trower NK, Bergdall AR, Dehmer SP, Maciosek MV (2016). A substudy evaluating treatment intensification on medication adherence among hypertensive patients receiving home blood pressure telemonitoring and pharmacist management. J Clin Pharm Ther.

[10] Abel N, Contino K, Jain N, Grewal N, Grand E, Hagans I (2015). Eighth joint national committee (JNC-8) guidelines and the outpatient management of Hypertension in the African-American population. North Am J Med Sci.

[11] Godbole A (2006). Patient adherence to Medical Treatment regimens: bridging the gap between behavioral Science and Biomedicine. Psychiatr Serv.

[12] Sanz EJ (2005). Patient adherence to medical treatment regimens: bridging the gap between behavioral science and biomedicine. BMJ.

[13] Baune BTh, Aljeesh Y, Bender R (2005). Factors of non-compliance with the therapeutic regimen among hypertensive men and women: a case-control study to investigate risk factors of Stroke. Eur J Epidemiol.

[14] Peerson A, Saunders M (2009). Health literacy revisited: what do we mean and why does it matter?. Health Promot Int.

[15] Marciano L, Camerini A-L, Schulz PJ (2019). The role of health literacy in Diabetes knowledge, self-care, and glycemic control: a meta-analysis. J Gen Intern Med.

[16] Marmot M, Friel S, Bell R, Houweling TA, Taylor S, Commission on Social Determinants of Health (2008). Closing the gap in a generation: health equity through action on the social determinants of health. The Lancet.

[17] Berkman ND, DeWalt DA, Pignone MP, Sheridan SL, Lohr KN, Lux L, et al. Literacy and health outcomes: summary. AHRQ Evid Rep Summ; 2004. PMC4781151 15819598

[18] La Vonne AD, Zun LS (2008). Assessing adult health literacy in urban healthcare settings. J Natl Med Assoc.

[19] Kindig DA, Panzer AM, Nielsen-Bohlman L. Health literacy: a prescription to end confusion. 2004. 25009856

[20] Selic P, Svab I, Repolusk M, Gucek NK (2011). What factors affect patients’ recall of general practitioners’ advice?. BMC Fam Pract.

[21] Visscher BB, Steunenberg B, Heijmans M, Hofstede JM, Devillé W, van der Heide I (2018). Evidence on the effectiveness of health literacy interventions in the EU: a systematic review. BMC Public Health.

[22] Tan JP, Cheng KKF, Siah RC-J (2019). A systematic review and meta-analysis on the effectiveness of education on medication adherence for patients with Hypertension, hyperlipidaemia and Diabetes. J Adv Nurs.

[23] Lor M, Koleck TA, Bakken S, Yoon S, Dunn Navarra A-M (2019). Association between Health Literacy and Medication adherence among hispanics with Hypertension. J Racial Ethn Health Disparities.

[24] Stokes K, Oronti B, Cappuccio FP, Pecchia L (2022). Use of technology to prevent, detect, manage and control Hypertension in sub-saharan Africa: a systematic review. BMJ Open.

[25] Inglis SC, Clark RA, Dierckx R, Prieto-Merino D, Cleland JGF (2015). Structured telephone support or non-invasive telemonitoring for patients with Heart Failure. Cochrane Database Syst Rev.

[26] Duchesne S, McMaugh A. Educational psychology for learning and teaching. Cengage AU; 2018.

[27] Nutbeam D, McGill B, Premkumar P (2018). Improving health literacy in community populations: a review of progress. Health Promot Int.

[28] Tran D, Coleman L, Lederer H, Miller B, Stanford M, Layson-Wolf C (2015). Development and evaluation of a hypertension-focused Educational Program Taught collaboratively by a registered Dietitian and a pharmacist. J Fam Consum Sci.

[29] Beaton DE, Bombardier C, Guillemin F, Ferraz MB (2000). Guidelines for the process of cross-cultural adaptation of self-report measures. Spine.

[30] Parker RM, Baker DW, Williams MV, Nurss JR (1995). The test of functional health literacy in adults. J Gen Intern Med.

[31] Osborn CY, Weiss BD, Davis TC, Skripkauskas S, Rodrigue C, Bass PF (2007). Measuring adult literacy in health care: performance of the newest vital sign. Am J Health Behav.

[32] Javadzade SH, Sharifirad Gh, Reisi M, Tavassoli E, Rajati F (2013). Health Literacy among adults in Isfahan, Iran. HSR.

[33] Lenz ER. Measurement in nursing and health research. Springer publishing company; 2010.

[34] Lawshe CH (1975). A quantitative approach to content validity. Pers Psychol.

[35] Rajati F, Sharifiebad T, Tavakol K, Almasi A, Karami S, Jamshidi HS (2021). Psychometric properties of the Persian Version of Cardiovascular Management self-efficacy scale. J Cardiovasc Nurs.

[36] Sadegh SS, Saadat PK, Sepehri MM, Assadi V (2018). A framework for m-health service development and success evaluation. Int J Med Inf.

[37] Carey RM, Whelton PK, ACC/AHA Hypertension Guideline Writing Committee. 2017. Prevention, Detection, Evaluation, and Management of High Blood Pressure in Adults: Synopsis of the 2017 American College of Cardiology/American Heart Association Hypertension Guideline. Ann Intern Med. 2018;168:351–8. 10.7326/M17-3203 29357392

[38] Institute of Medicine (US) (2004). Committee on Health Literacy. Health literacy: a prescription to end confusion.

[39] Nooshin Peyman F, Behzad A, Taghipoor H, Esmaeli (2016). Assessment of the Effect of a Health Literacy Educational Program for Health Personnel on promoting self-efficacy among patients with chronic Diseases. Mui-Jhsr.

[40] Zhuang R, Xiang Y, Han T, Yang G-A, Zhang Y (2016). Cell phone–based health education messaging improves health literacy. Afr Health Sci.

[41] Sharifirad G, Mostafavi F, Hasanzade A, Javadzade S, Radjati F, Reisi M (2012). Relationship between health literacy, health status, and healthy behaviors among older adults in Isfahan, Iran. J Educ Health Promot.

[42] Mosleh SM, Almalik MM (2016). Illness perception and adherence to healthy behaviour in Jordanian coronary Heart Disease patients. Eur J Cardiovasc Nurs.

[43] Kalantari H, Bagherian Sararoodi R, Afshar H, Khoramian N, Forouzandeh N, Daghagh Zadeh H (2012). Relationship between Illness perceptions and quality of life in patients with irritable bowel syndrome. J-Mazand-Univ-Med-Sci.

[44] Rajpura J, Nayak R (2014). Medication adherence in a sample of Elderly suffering from Hypertension: evaluating the influence of Illness perceptions, treatment beliefs, and Illness Burden. J Manag Care Pharm.

[45] Chen S-L, Tsai J-C, Chou K-R (2011). Illness perceptions and adherence to therapeutic regimens among patients with Hypertension: a structural modeling approach. Int J Nurs Stud.

[46] Ross S, Walker A, MacLeod MJ (2004). Patient compliance in Hypertension: role of Illness perceptions and treatment beliefs. J Hum Hypertens.

[47] Kang CD, Tsang PPM, Li WTL, Wang HHX, Liu KQL, Griffiths SM (2015). Determinants of medication adherence and blood pressure control among hypertensive patients in Hong Kong: a cross-sectional study. Int J Cardiol.

[48] Altin SV, Stock S (2016). The impact of health literacy, patient-centered communication and shared decision-making on patients’ satisfaction with care received in German primary care practices. BMC Health Serv Res.

[49] Kilic HFPD, Dag RN, MSc S (2020). The relationship between Health Literacy and Medication Adherence in a Hypertensive Patient Population. Int J Caring Sci.

[50] Wannasirikul P, Termsirikulchai L, Sujirarat D, Benjakul S, Tanasugarn C (2016). Health literacy, medication adherence, and blood pressure level among hypertensive older adults treated at primary health care centers. Southeast Asian J Trop Med Public Health.

[51] Hallberg I, Ranerup A, Kjellgren K (2016). Supporting the self-management of Hypertension: patients’ experiences of using a mobile phone-based system. J Hum Hypertens.

[52] Rajati F, Feizi A, Tavakol K, Mostafavi F, Sadeghi M, Sharifirad G. Comparative Evaluation of Health-Related Quality of Life Questionnaires in Patients With Heart Failure Undergoing Cardiac Rehabilitation: A Psychometric Study. Arch Phys Med Rehabil. 2016;97:1953–62. 10.1016/j.apmr.2016.05.010 27288709

